# Harnessing Bulk‐Segregant Mapping to Identify Trait‐Associated Genes in the Allopolyploid Model Plant *Nicotiana benthamiana*

**DOI:** 10.1111/pbi.70560

**Published:** 2026-01-29

**Authors:** Zuba Ahmed, Jiyuan An, Satomi Hayashi, Julia Bally, Chris Winefield, Peter M. Waterhouse

**Affiliations:** ^1^ Centre for Agriculture and the Bioeconomy, School of Biology and Environmental Sciences Queensland University of Technology Brisbane City Queensland Australia; ^2^ ARC Centre of Excellence for Plant Success in Nature and Agriculture Queensland University of Technology Brisbane City Queensland Australia; ^3^ Department of Wine Food and Molecular Biosciences, Faculty of Agriculture and Life Sciences Lincoln University, Te Whare Wānaka o Aoraki Lincoln New Zealand

**Keywords:** *AcMYB110*, anthocyanin biosynthesis, bulk segregant analysis, forward genetics, mapping by sequencing, *Nicotiana benthamiana*, RNAseq

## Abstract

Forward genetics has been instrumental in identifying genes underlying desirable traits, yet its application to polyploid plants, many of which are key agricultural crops, remains challenging due to their genomic complexity. Therefore, we developed BenthMap, a bulk segregant analysis platform for high‐throughput trait mapping and gene discovery, in the allotetraploid model plant *Nicotiana benthamiana*. BenthMap leverages high‐quality genome assemblies of two genetically and phenotypically distinct strains, LAB and QLD. To validate the pipeline, we investigated their contrasting anthocyanin responses. Transient overexpression of *AcMYB110*, an activation regulator of anthocyanin biosynthesis, induces robust anthocyanin production in QLD leaves but gives a detrimental, often necrotic, response in LAB. Using BenthMap and a population derived from selfing the F1 hybrid of a LAB × QLD cross (F1S1 population), with genome coverage as low as 10×, we mapped the necrotic LAB response to a 3.5 Mb homozygous region on chromosome 10. This region contains a leucoanthocyanidin dioxygenase gene. Transiently expressing the QLD version of this gene, along with *AcMYB110*, restored robust anthocyanin accumulation in LAB, validating the causal gene. These findings demonstrate BenthMap's utility for rapid trait‐gene identification in *N. benthamiana* and have potential for application to other allopolyploid plants.

## 1 Introduction

Forward genetics has proved to be a powerful approach for genetic mapping of desirable traits or mutations. The *Arabidopsis thaliana* ecotypes, Landsberg *erecta* (L*er*) and Columbia‐0 (Col‐0), served as foundational models, driving the development of bulk segregant analysis and mapping‐by‐sequencing, which combine next‐generation sequencing (NGS) with classical genetic mapping to identify causal loci (Schneeberger et al. 2009; Austin et al. 2011; James et al. 2013; Allen et al. 2013; Klein et al. 2018). A significant advancement in this field was the computational pipeline SHOREmap, enabling the analysis of NGS data from F2 mapping populations to detect regions of homozygosity shared with the mutant parent, thereby pinpointing phenotype‐governing mutations (Schneeberger et al. 2009). While these tools have been extensively applied in diploid model systems, their adaptation to polyploid species has remained limited (Schneeberger et al. 2009). A notable exception was the adaptation of a SHOREmap‐like pipeline to hexaploid wheat, which successfully mapped the *Yr7* locus conferring yellow rust resistance (Gardiner et al. 2014), demonstrating the potential of forward genetics in polyploid crops. Despite these advances, functional validation of candidate genes identified through forward genetic approaches in hard‐to‐transform crop species can be a laborious and resource‐intensive process (Tyurin et al. 2020).

*Nicotiana benthamiana* (‘LAB’ strain) has emerged as a unique model plant widely employed in reverse genetics (Bally et al. 2018). Its amenability to transient transformation using *Agrobacterium*‐mediated infiltration enables virus‐induced gene silencing (VIGS) or rapid gene expression, approaches that work poorly in other model species such as *Arabidopsis* (Goodin et al. 2008). This offers an exceptional system for engineering metabolic pathways, understanding gene function and production of biopharmaceutical proteins (Ranawaka et al. 2023). Despite its prominence in reverse genetics, *N. benthamiana* has been underutilised for forward genetics, with the notable exception of the EMS mutant screen conducted by Schultink et al. (2019). This limited adoption is primarily due to its large, complex and repetitive allopolyploid genome, which complicates mapping approaches.

The LAB strain, which has been used globally, comes from a single source (Goodin et al. 2008; Bally et al. 2015). However, recent studies have illustrated broader diversity among wild *N. benthamiana* populations (Ranawaka et al. 2023). In particular, accessions from Queensland (QLD) and Northern Territory (NT) exhibit distinct phenotypes and the ability to produce and accumulate anthocyanins. While LAB is commonly used in anthocyanin production studies, it shows poor pigment accumulation when the pathway is induced (Thole et al. 2019). In contrast, QLD and NT can robustly produce anthocyanin in their leaves (Ranawaka et al. 2023), such that the NT accession has been used in several anthocyanin‐related studies (Thole et al. 2019; Albert et al. 2021; Plunkett et al. 2018; Lafferty et al. 2022; Nguyen et al. 2023; Dare et al. 2024; Moss et al. 2024). The recent availability of high‐quality chromosome‐level genome assemblies for both LAB and QLD has significantly enhanced the prospect of NGS‐based trait mapping in *N. benthamiana* (Ranawaka et al. 2023). These comprehensive genomic resources provide the necessary foundation for the precise identification of genetic variants and accurate mapping of phenotypic traits. Furthermore, the evolutionary history of *N. benthamiana* accessions represents a rich source of genetic variation that can be explored using forward genetic approaches. The QLD accession, in particular, serves as a valuable source of genetic diversity, exhibiting distinctive traits such as its distinct virus resistance (Hayashi et al. 2024) and survival strategy in drought conditions (Asadyar et al. 2024). These natural variations offer opportunities to uncover novel alleles and elucidate the genetic architecture underlying complex traits, potentially informing crop improvement strategies across *Solanaceae* species. Additionally, the ability of these accessions to inter‐cross and produce fertile offspring further establishes *N. benthamiana* as a valuable genetic model, providing similar advantages to *A. thaliana* . Comparative genomic analysis between LAB and QLD is not only useful for identifying novel alleles but also for gaining insights into the evolution of the *Suaveolentes* section of *Nicotianas*. The system of contrasting anthocyanin production seemed a good candidate for testing forward genetics in the large reference genome of *N. benthamiana*, and if successful, to identify the genes responsible for robust anthocyanin production.

Anthocyanin biosynthesis is one of the most extensively studied plant metabolic pathways, with a specific sub‐group (sub‐group 6) of R2R3 MYB transcription factors (TFs) serving as key activation regulators (Liu et al. 2018). Overexpression of such an R2R3 MYB TF, *AcMYB110* from kiwifruit (Montefiori et al. 2015), results in substantial anthocyanin pigment accumulation in QLD leaf but is detrimental in LAB. Anthocyanin production in *N. benthamiana* leaf tissue is an excellent reporter system for studying gene expression due to its visible phenotype and well‐characterised biosynthetic pathway (Nguyen et al. 2023; Bond et al. 2016). The pathway is highly conserved among plant species, but differences in gene copy number and presence/absence variations contribute to contrasting pigmentation across species and ecotypes (Su et al. 2019; Lu et al. 2021). Investigating such differences between accessions using conventional comparative transcriptomics in recent allopolyploid species can be challenging due to mapping bias among similar homeologous genes (Coombes et al. 2024). To examine this limitation in the more ancient aneu‐allotetraploid *N. benthamiana*, we employed bulk segregant analysis (BSA) and mapping‐by‐sequencing in our newly developed workflow BenthMap, which incorporates the homozygosity scoring algorithm originally developed by Gardiner et al. (2014), as an unbiased approach to investigate the contrasting anthocyanin response observed between LAB and QLD.

Here, we demonstrate that using a combined approach of BenthMap with RNA sequencing (RNAseq), in a segregating natural population derived from a *N. benthamiana* LAB × QLD cross, we were able to uncover the genetic basis of differential anthocyanin production. This approach revealed a premature stop codon and four amino acid changes in the leucoanthocyanidin dioxygenase (*LDOX*) gene of chromosome 10 (Ch10) in LAB, accounting for its incompetence in anthocyanin accumulation. Analysing the genomes, we further identified two other non‐functional copies of *LDOX* in both LAB and QLD. These findings not only elucidate a key component of the anthocyanin biosynthetic pathway in *N. benthamiana* but also underscore the potential utility of this system for future forward genetic studies in this and other complex allopolyploid plants.

## 2 Results

### 2.1 QLD Exhibits an Accelerated Transcriptional Response Compared to LAB Following Anthocyanin Pathway Induction

**FIGURE 1:**
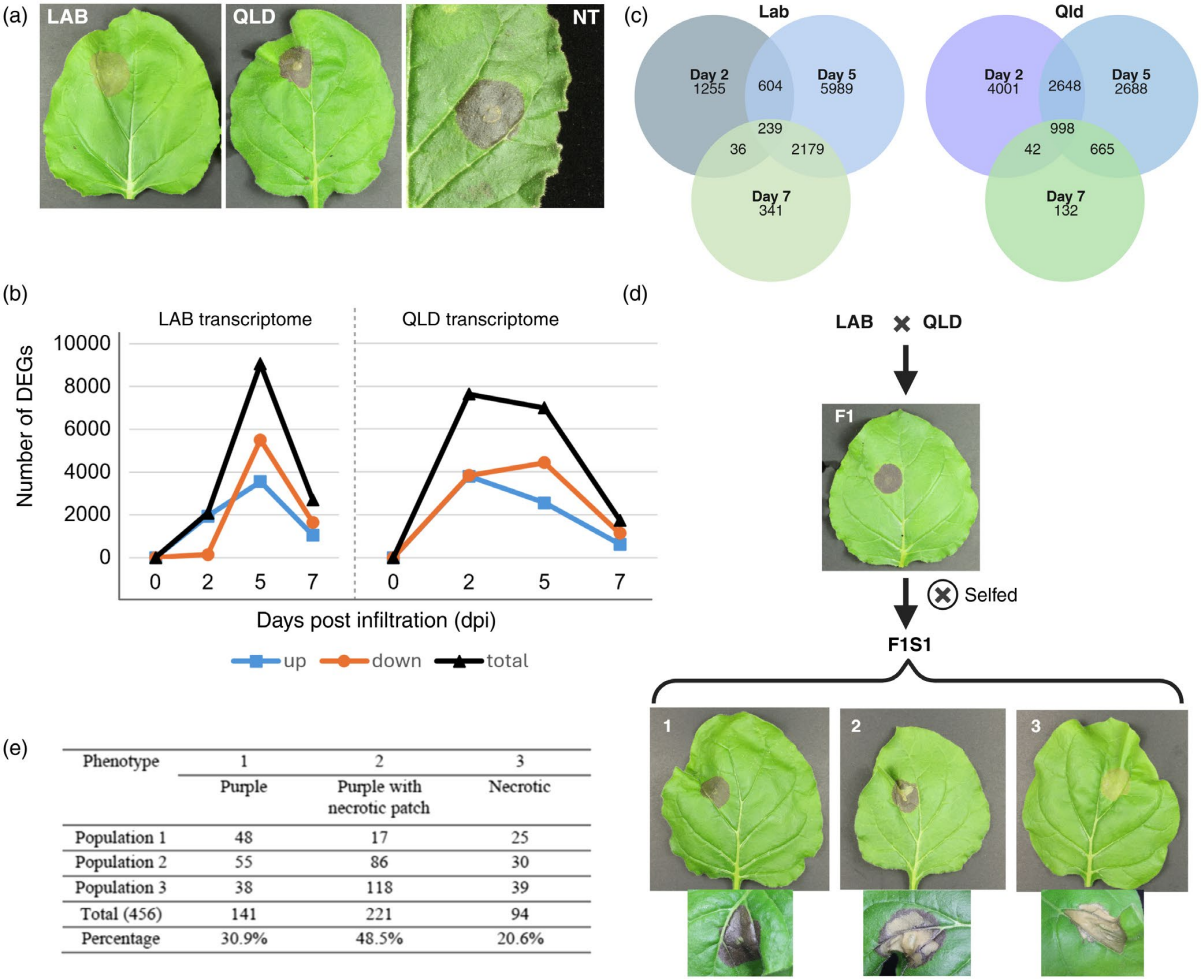
*AcMYB110* overexpression in *N. benthamiana* leaves. (a) Phenotype at 5 days post‐infiltration in LAB, QLD and NT accessions. (b) Number of significantly differentially expressed genes in LAB (left) and QLD (right) over the three time‐points post *AcMYB110* infiltration, showing total number of DEGs (black line), number of upregulated (blue line) and downregulated (orange line) genes. (c) Venn diagrams displaying comparisons between the number of shared significantly differentially expressed genes (*p*‐value < 0.001) at three time‐points post infiltration (Day 2, 5 and 7) in LAB (reads aligned to the LAB transcriptome) and QLD (reads aligned to the QLD transcriptome). (d) Phenotype at 5 days post‐infiltration in LAB × QLD F1 and F1S1 individuals. (e) Number of plants exhibiting three distinct phenotypes: (1) purple, (2) purple with necrotic patch and (3) necrotic, among three F1S1 populations.

To re‐examine the reported problems with anthocyanin accumulation in LAB (Thole et al. 2019; Albert et al. 2021; Grützner et al. 2024), *AcMYB110* was overexpressed in their leaf tissue via agroinfiltration, and the resulting phenotype was compared to that of QLD and NT. Within 5 days post‐infiltration (dpi), the infiltrated patches became necrotic in LAB, supporting prior findings, while deep purple patches were observed on the corresponding leaves of QLD and NT (Figure 1a). A previous study by Bond et al. (2016) identified 2462 genes, including many anthocyanin biosynthetic and regulatory genes, to be upregulated in LAB leaf upon agroinfiltration of R2R3 transcription factor *AcMYB10*. These genes possibly included those responsible for the differential anthocyanin responses to pathway induction observed in LAB and QLD. To investigate further, LAB and QLD were similarly infiltrated with either 35S::*AcMYB110* or a modified dysfunctional version of the *AcMYB110* construct (Control; Figure S1), and changes in global gene expression were examined by RNAseq at three different time points: (1) before visible pigment accumulation (2 dpi), (2) at first observable purple pigmentation in QLD (5 dpi), and (3) at the highest pigmentation in QLD (7 dpi). The use of dysfunctional *AcMYB110* agroinfiltration as control was employed to eliminate confounding differential expression effects from *Agrobacterium* infection and associated T‐DNA transfer. Many genes were more rapidly induced in QLD than in LAB. A peak in the number of upregulated genes was observed at 2 dpi for QLD, with approximately 3700 upregulated genes, compared to only about half that number in LAB (Figure 1b, Figure S3). The peak number of upregulated genes in LAB was observed at 5 dpi, with ~3500 genes upregulated, while QLD showed slightly fewer genes upregulated (~2500 genes). Interestingly, QLD not only exhibited rapid gene induction, but also a rapid response in gene repression with a similar number of genes upregulated and downregulated at 2 dpi (Figure 1b and Figure S3). For both LAB and QLD, the number of both up‐ and down‐regulated genes was significantly lower by 7 dpi compared to the earlier timepoint (5 dpi) (Figure 1b,c). These differentially regulated genes included regulatory and transporter genes, and genes involved in the anthocyanin biosynthetic pathway and the general phenylpropanoid pathways (Figure S2). In concordance with the data from Bond et al. (2016), the expression profiles of LAB and QLD were generally similar except for the timing of induction (Figure S4). Nevertheless, we were unable to identify specific differences between LAB and QLD that pointed to the gene(s) responsible for their distinct anthocyanin‐related responses, encouraging us to investigate a bulk segregant analysis approach for the identification of causal gene(s).

### 2.2 A Single Recessive Gene Is Linked to the Deficient Anthocyanin Response in LAB

The availability of the chromosome‐level QLD reference genome sequence motivated the use of a LAB × QLD cross, rather than LAB × NT. To confirm preliminary findings of 3:1 segregation of anthocyanin competence in LAB × QLD crosses (Ranawaka et al. 2023), and to better understand the nature of the gene responsible for successful anthocyanin production in QLD, three F1 selfed (F1S1) populations with a total of 456 individuals were screened for their anthocyanin response (Figure 1d,e). Three distinct phenotypes were observed: purple, purple with necrosis in the centre and fully necrotic infiltration patches (Figure 1d). Populations 1 and 3 showed a segregation ratio of 3:1, with non‐necrotic phenotypes (purple and purple with some necrosis) outnumbering the necrotic phenotype (population 1: *X* ^2^ = 0.37, *p* = 0.54, population 3: *X* ^2^ = 2.60, *p* = 0.107), suggesting that the necrotic phenotype is controlled by a single recessive gene (Figure 1d). With the difficulty in distinguishing the purple phenotypes displayed by the homozygous dominant and the heterozygous individuals, we decided to use pooled DNA representative of plants showing the necrotic phenotype (presumably homozygous recessive) for bulk segregant analysis.

### 2.3 BenthMap Workflow Highlights a Candidate Genomic Region on QLD Chromosome 10

**FIGURE 2:**
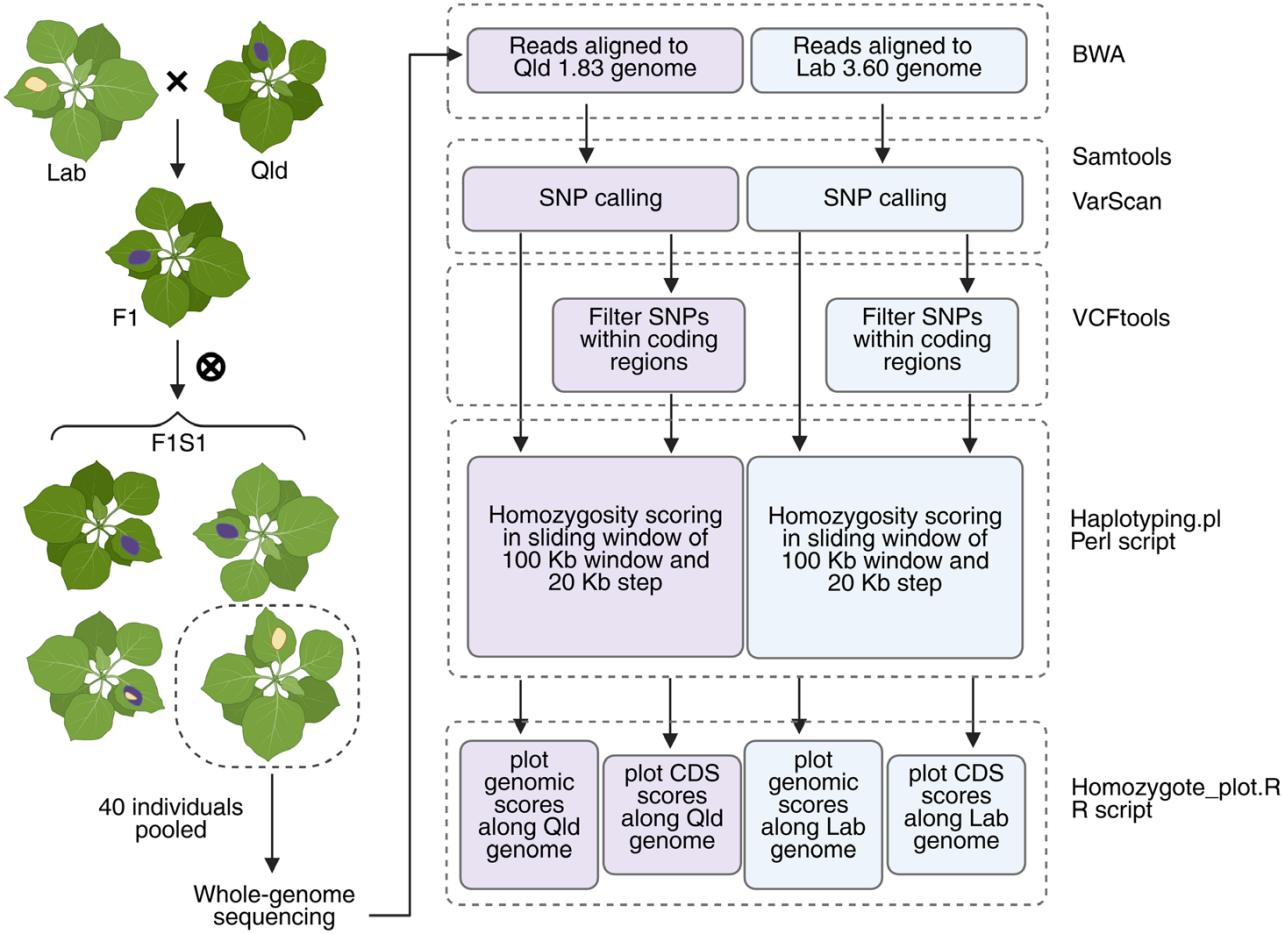
BenthMap workflow for mapping of the causal locus in *N. benthamiana*. F1S1 reads were mapped to LAB and QLD genomes, followed by parallel bioinformatic pipelines to determine allele frequencies specific to either LAB or QLD parents.

Sequencing a single pool of genomic DNA from 40 F1S1 individuals (Figure 2) with the induced necrotic phenotype produced 1 894 567 550 high quality paired‐end reads (Q20 ≥ 99.99% and Q30 ≥ 88.62%) (Table S2). This equated to approximately 202‐fold (200×) genome coverage of the LAB (2.75 Gb) and/or QLD (2.72 Gb) genomes with mapping rates of 99.86% and 99.78%, respectively (Table S3). After removing single nucleotide polymorphisms (SNPs) where the alternate base appeared in less than 30% of reads, a total of 28 804 681 and 28 619 735 SNPs were identified from F1S1 reads mapping to the LAB and QLD genomes, respectively. These were further filtered to retain only those located within coding regions, reducing the SNPs to 1 733 898 for LAB and 1 784 095 for QLD (Table S4). SNP variations between the LAB and QLD parental genomes were investigated using RNAseq data from parental wild type tissue, revealing that QLD retained approximately four times more heterozygosity than LAB (Table S10).

**FIGURE 3:**
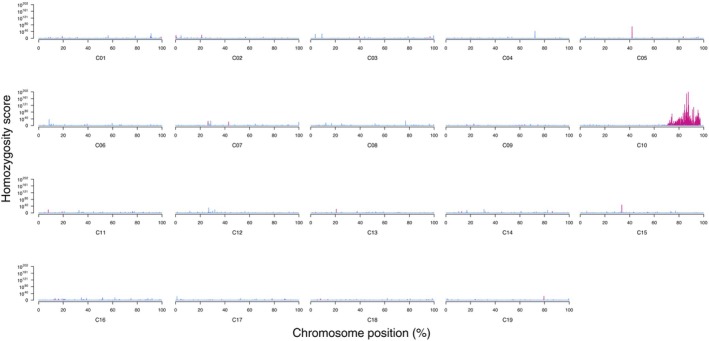
Homozygosity scores from F1S1 sequence reads mapped to the *N. benthamiana* genome. Reads mapped to LAB (blue line) or the QLD (pink line) genomes and filtered to retain SNPs within coding regions. The *x*‐axis represents a percentage scale of the actual chromosome lengths.

To identify genomic regions associated with the necrotic phenotype, we applied the BenthMap workflow (Figure 2), which emulates the principles of SHOREmap (Schneeberger et al. 2009), incorporating the improvements by Gardiner et al. (2014) and uses a sliding 100 Kb window along the length of each chromosome of LAB and QLD, and an optimised step size of 20 Kb, to analyse the number of consecutive homozygous SNPs. The SNPs within the coding sequence regions for LAB and QLD were filtered using genome annotations, which removes ~93% of non‐informative SNPs. A homozygous SNP was defined as having > 80% of the reads with an alternative base. The reads for most individual SNPs were present in approximately equal proportions (i.e., ~50% for LAB and QLD). However, 31 119 SNPs were classified as homozygous for the QLD alleles, and the BenthMap‐calculated homozygosity scores for each chromosome revealed that most contributed to a strong peak on QLD Ch10 (Figure 3). The peak encompasses a 39 Mb region from position ~100 000 000 to the end of the chromosome (139635422) and contains 767 annotated genes.

### 2.4 Determining the Optimal and Minimal Sequence Depth of F1S1 Reads Required to Resolve the Mapping Region

**FIGURE 4:**
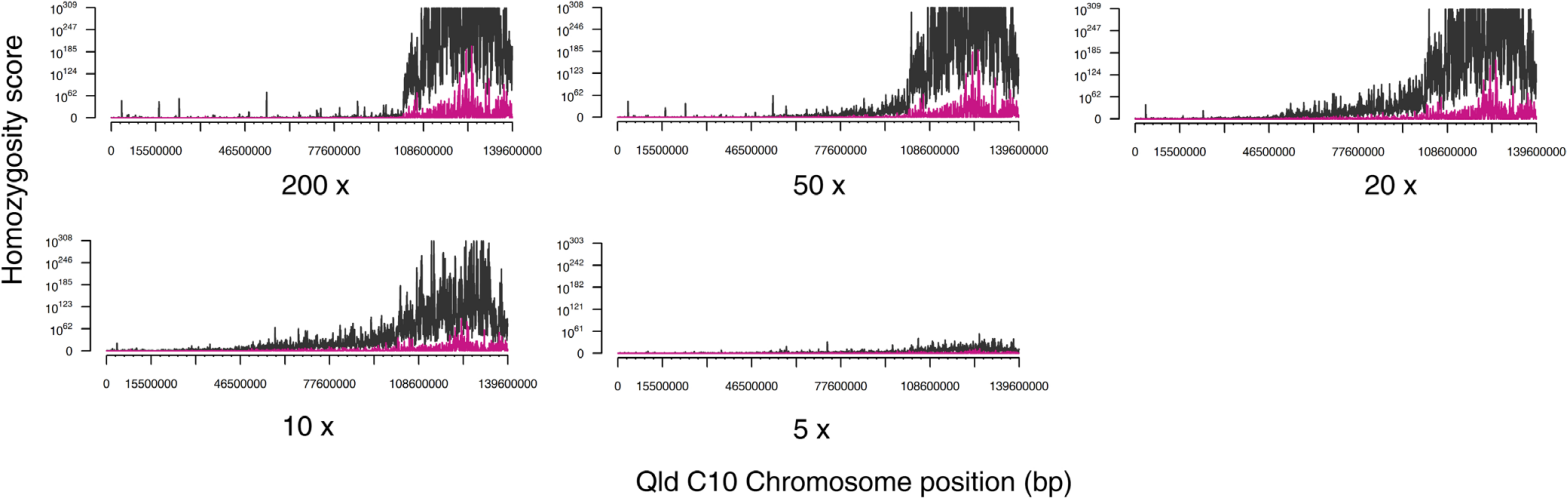
Homozygosity scores in QLD chromosome 10 for representative sub‐sets of F1S1 reads. Homozygosity scores were calculated for representative sub‐sets of F1S1 reads, to achieve 50×, 20×, 10× and 5× coverage of the *N. benthamiana* genome. Scores calculated for all SNPs across chromosome 10 (dark grey line) and for SNPs within coding regions only (pink line).

To investigate the minimum sequencing depth that would reliably detect this trait‐associated region, we down‐sampled the raw F1S1 fastq reads using S‐leaping software (Kuwahara and Gao 2023) to generate sub‐sets of 50×, 20×, 10× and 5× coverage (Table S5). Analysing these with BenthMap showed that even at 10× coverage, either for coding‐region‐specific or whole genome SNPs, the 39 Mb region of homozygosity on QLD Ch10 was readily detected. However, the use of whole genome SNPs resulted in a dramatically higher peak beyond the maximum threshold value (Figure 4).

### 2.5 Fine Mapping Reveals a 3.5 Mb Region Containing a Leucoanthocyanidin Dioxygenase ( *LDOX* ) Gene

**FIGURE 5:**
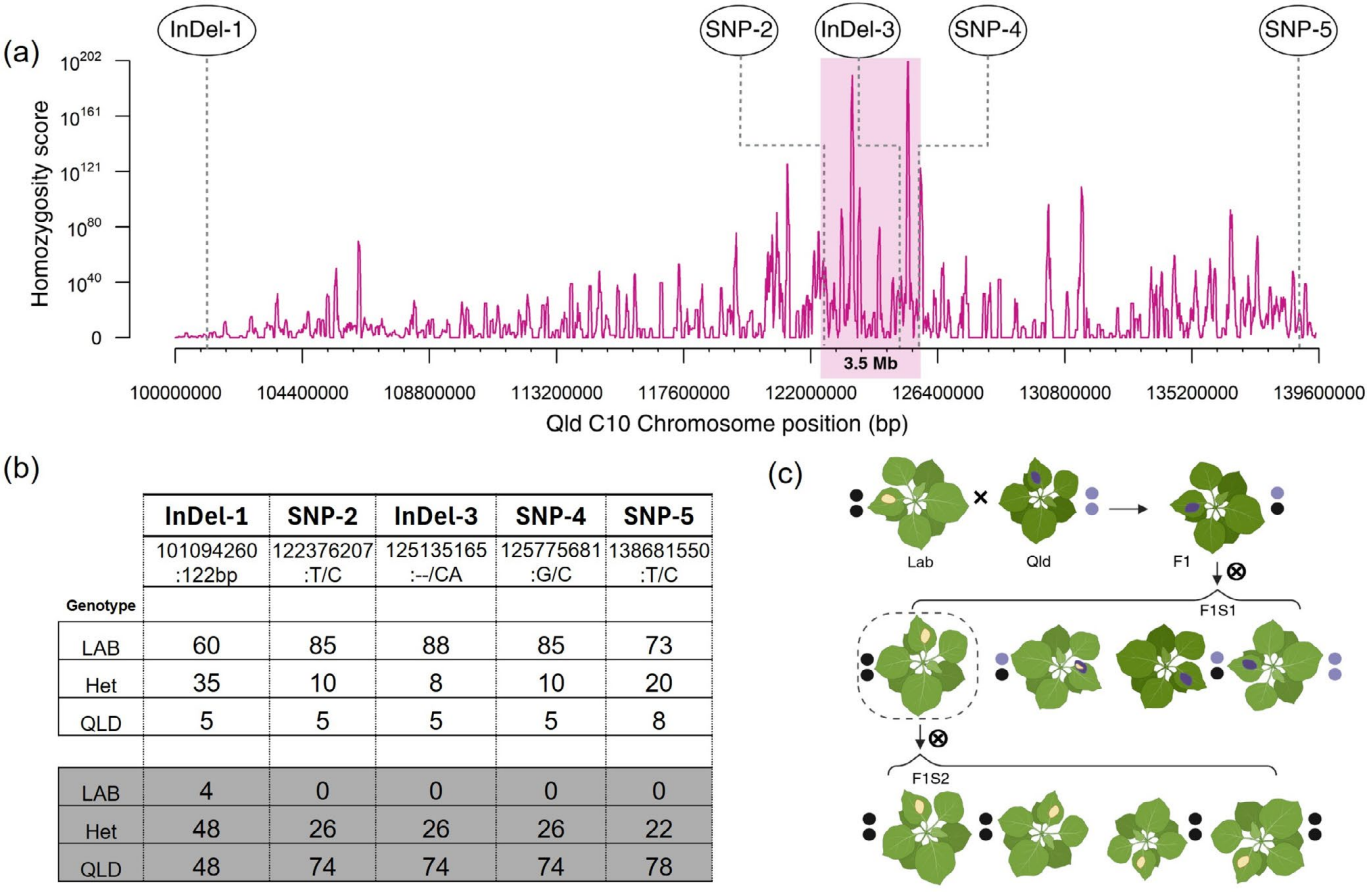
Fine mapping of the causal gene using molecular markers. (a) Position of the 5 markers along the 39 Mb region of QLD Ch10 and the 3.5 Mb region with the highest peaks (pink shade). (b) Genotyping results of the 5 marker positions for F1S1 plants with necrotic phenotype (*n* = 40, top, no shading), and purple phenotype (*n* = 23, bottom, grey shaded). Percentage of individuals exhibiting homozygous LAB (LAB), Heterozygous (Het) and homozygous QLD (QLD) genotypes are displayed for each marker position. (c) Predicted genotypes of LAB, QLD, F1S1 and F1S2 (progeny of necrotic phenotype F1S1 individuals, *n* = 56) at causal loci (displayed as black circle for LAB allele in and light purple circle for QLD allele).

To validate that the 39 Mb candidate region was responsible for the anthocyanin robustness in QLD and the detrimental response in LAB, we studied the inheritance of five diagnostic loci (InDel‐1, SNP‐2, InDel‐3, SNP‐4 and SNP‐5), each characterised with either a large (> 100 bp) insertion or deletion (InDel), or a SNP suitable for restriction fragment length polymorphism analysis (Figure 5a, Table S8). SNP‐2, InDel‐3 and SNP‐4 are located within a 3.5 Mb region harbouring the highest signal for homozygosity, and InDel‐1 and SNP‐5 are 15–20 Mb upstream and downstream of this refined region, respectively. The genotypes of these 5 loci were analysed for 63 F1S1 individuals, 40 of which had the inducible necrotic phenotype, and 23 had the purple phenotype. More than 60% of the plants with the inducible necrotic phenotype were homozygous for the LAB allele at all five loci, while more than 85% of them were homozygous for LAB at SNP‐2, InDel‐3 and SNP‐4 located at the 3.5 Mb refined region (Figure 5). In contrast, none of the individuals with the purple phenotype were homozygous for the LAB allele at these loci. Eight out of the 40 anthocyanin incompetent F1S1 individuals were self‐fertilised to produce F1S2 individuals. When tested for anthocyanin production, all F1S2 plants arising from the 8 anthocyanin incompetent F1S1 parents displayed the necrotic phenotype (*n* = 56). This result confirmed that the parental F1S1 individuals were 100% homozygous for the LAB allele at the loci harbouring the causal gene. Collectively, this strongly suggested that the genomic region predicted by BenthMap underlies the differential anthocyanin response.

**FIGURE 6:**
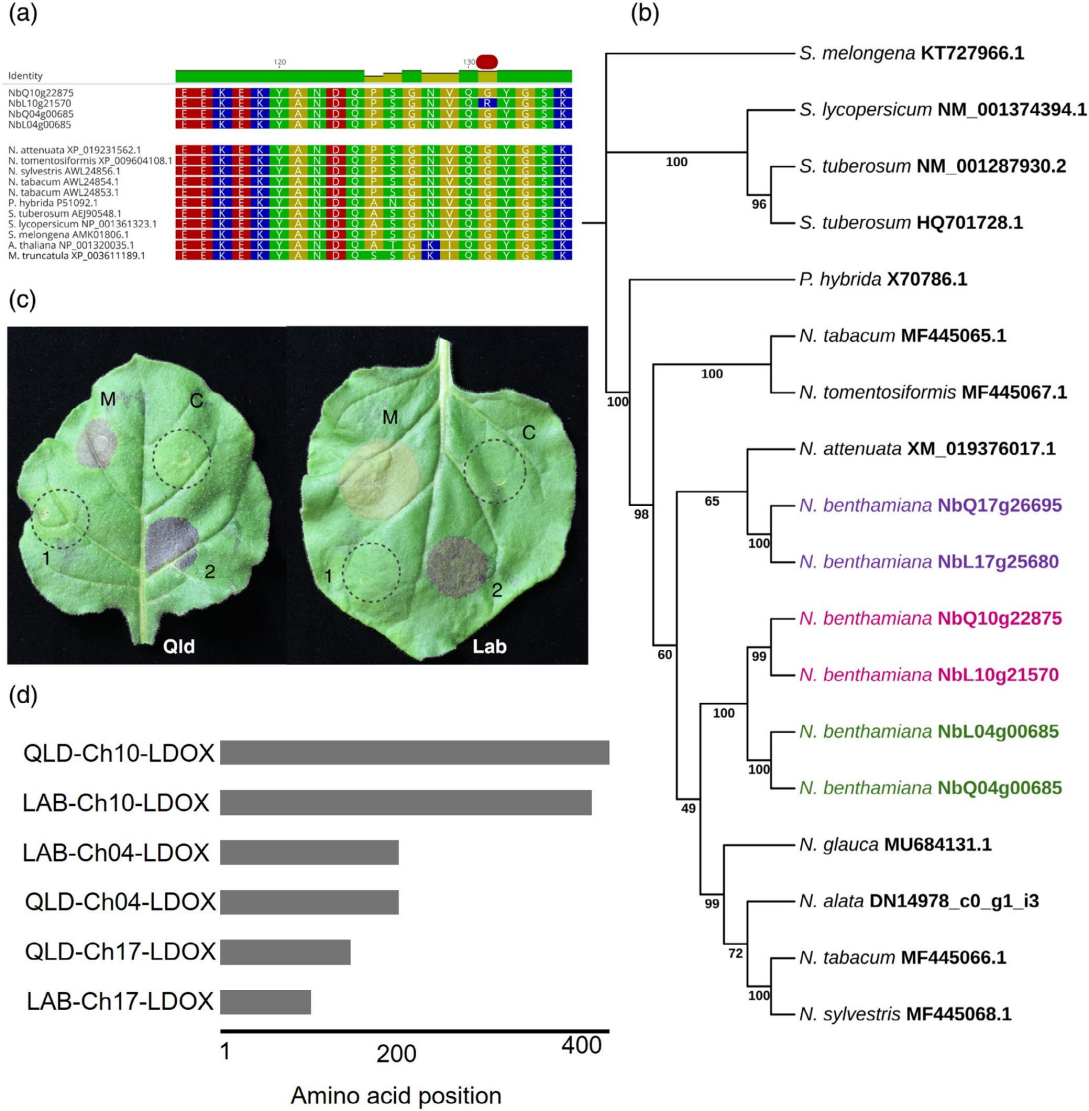
Sequence analysis and functional characterisation of LDOX proteins in *N. benthamiana*. (a) Sequence alignment of LDOX proteins highlighting the Glycine (G; yellow) to Arginine (R; purple) mutation at position 131 in LAB‐Ch10‐LDOX (NbL10g21570) (red highlight). (b) Maximum‐likelihood cladogram of *LDOX* nucleotide sequences including the six *N. benthamiana* LAB (NbL) and QLD (NbQ) LDOXs and *LDOX*/*ANS* from selected *Solanaceae* species (*Solanum tuberosum, Solanum lycopersicum, Solanum melongena, Nicotiana tomentosiformis, Nicotiana tabacum, Nicotiana sylvestris, Nicotiana attenuata, Nicotiana alata, Nicotiana glauca and Petunia hybrida*). Genbank accession numbers for the sequence used are listed next to species names except for *N. alata* , *N. glauca* and *N. benthamiana* for which sequence IDs correspond to their genome annotations. The tree was generated using IQ‐TREE using the TIM2 + F + G4 substitution model, and branch labels represent ultrafast bootstrap support (%). The cladogram is unrooted however, *S. melongena* is drawn at the root. (c) Effect of transient co‐expression of *QLD‐Ch10‐LDOX* and *AcMYB110* on anthocyanin accumulation in LAB and QLD leaf. Pictured at 5 days after infiltration with 35S::*AcMYB110* (M), pS191 (empty vector control) (C), mixture of pS191+ 35S::*QLD‐Ch10‐LDOX* (1) and mixture of 35S::*AcMYB110* + 35S::*QLD‐Ch10‐LDOX* (2). (d) Structure of *N. benthamiana* LDOX proteins in reference to QLD‐Ch10‐LDOX.

This 3.5 Mb trait‐associated region in the QLD genome contains 111 annotated genes, of which two genes, a *leucoanthocyanidin dioxygenase* (*LDOX*; NbQ10g22875; NbL10g21570) and the transcription factor *EGL1* (NbQ10g21850; NbL10g20620), a basic helix–loop–helix (bHLH) orthologue of *NtJaf13b* (Montefiori et al. 2015), are associated with the anthocyanin biosynthesis pathway. Both were among the 35 genes in this region that showed differential expression in leaves agroinfiltrated with 35S::*AcMYB110* (Table S6). The *LDOX* gene was greatly upregulated (> 10 fold) at all three timepoints (2, 5 and 7 dpi) in both QLD and LAB, whereas the expression of *EGL1* was slightly downregulated in QLD at 5 dpi (Tables S6 and S7). Further sequence analysis revealed a premature stop codon in NbL10g21570 (exon 2; position 1167 bp) resulting in a 388 amino acid (aa) LDOX protein, a protein shorter by 18 aa and approximately 31 aa, compared to that of QLD and other Solanaceous species (Nguyen et al. 2023; Wang et al. 2019; Jun et al. 2018), respectively (Figure 6d). However, the most striking difference was the G131R mutation in the N‐terminal domain of NbL10g21570, replacing the small polar glycine with a larger positively charged arginine in this highly conserved domain (Interpro domain: IPR026992) (Figure 6a; Figure S5).

### 2.6 Agroinfiltration of *NbQLDC10LDOX* Enables Robust Anthocyanin Production in LAB

To validate whether the *AcMYB110*‐induced necrotic phenotype in LAB is due to the form of the LDOX gene on Ch10, we agroinfiltrated LAB leaves with 35S::*AcMYB110* supplemented with either 35S::*QLD‐Ch10‐LDOX* or 35S::*LAB‐Ch10‐LDOX*. Expression of *QLD‐Ch10‐LDOX*, but not *LAB‐Ch10‐LDOX*, enabled LAB leaves to robustly produce anthocyanin (Figure 6c; Figure S7), confirming that this gene is responsible for the differential anthocyanin responses between LAB and QLD.

Examining the *N. benthamiana* genome for other *LDOX*‐like genes identified two additional copies in both QLD (NbQ17g26695 and NbQ04g00685) and LAB (NbL17g25680 and NbL04g00685). Comparing their nucleotide sequences with those from a range of *Solanaceae* species placed the Ch04 and Ch10 *LDOX*‐like genes in one group and the Ch17 *LDOX*‐like genes into a separate group (Figure 6b), suggesting the genes in each group may have originated from a different diploid ancestral parent. The two *N. tabacum LDOX* genes grouped separately with each of its two parental accessions, *N. tomentosiformis* and *N. sylvestris* (Schiavinato et al. 2020). Similarly, the Ch04/Ch10 *LDOX* genes of *N. benthamiana* grouped with the *N. sylvestris LDOX* gene, whereas the Ch17 *LDOX* grouped with the *N. attenuata* gene. This suggests that the *LDOX*‐like genes on Ch10 and Ch17 are likely homeologs, and those on Ch04 possibly arose by duplication from Ch10. All of the Ch04 and Ch17 LDOX genes were identified to contain premature stop codons, only coding for half or less of the full protein sequence (Figures 6d and S5, Tables S11 and S12). Unsurprisingly, overexpression of these *LDOX* along with *AcMYB110* failed to facilitate robust anthocyanin production in LAB leaves (Figure S7). This collective dysfunctionalisation of Ch04 and Ch17 LDOX‐like genes is probably due to the progressive diploidisation of *N. benthamiana's* allotetraploid genome (Ranawaka et al. 2023).

## 3 Discussion

*Arabidopsis thaliana* has a small and well documented genome and has been the mainstay of plant genetic research for more than 30 years. A recent enhancement of its utility has stemmed from the mapping pipelines SHOREmap and NGM, which, using the genome sequences of Col‐0 and L*er*, can rapidly identify phenotype‐governing genes. Over the past two decades, *N. benthamiana* has been increasingly employed for biotechnology and as a companion to *Arabidopsis* for functional genomics. It has also been used as a model plant for studying a number of metabolic pathways. However, much of the research uses *N. benthamiana* as a ‘blank canvas’ upon which to impose genes and pathways from other species rather than investigating, utilising and understanding its endogenous equivalents. The recent production of high quality, chromosome‐level, genome sequence assemblies of LAB and QLD (Ranawaka et al. 2023) raised the possibility of using a SHOREmap‐like approach to determine the genetic players conferring the plants' rich diversity of secondary metabolic pathways and to gain insights into their evolutionary significance (Vollheyde et al. 2023; Golubova et al. 2024).

*Nicotiana benthamiana*, with its allotetraploid genome, large intergenic regions and considerable inter‐subgenome recombination, presents challenges and opportunities distinct from *A. thaliana* . It also appears to be undergoing rapid diploidization with concomitant dysfunctionality of genes and pathways, especially in LAB (Ranawaka et al. 2023). The obvious visual difference in functionality of the anthocyanin biosynthesis pathway between LAB and QLD presented an opportunity to test whether a SHOREmap‐like approach could identify causal genes of such differences. The previous work by Bond et al. (2016) using a combination of agroinfiltration and RNAseq of LAB leaves identified many gene targets of the anthocyanin‐inducing transcription factor, *AcMYB10*. Using a similar R2R3 transcription factor (*AcMYB110*), we recapitulated the regulation of these targets in both LAB and QLD, but were unable to find transcriptomic differences that correlated with the contrasting LAB and QLD phenotypes. A major factor in this was the presence of dysfunctional gene copies scattered around their allotetraploid genomes; many of them have insertions, deletions and premature stop codons but are still transcribed and captured in short‐read RNAseq data.

The BenthMap workflow (Figure 2) clearly identified that a small region on Ch10 is responsible for the differing response, and transient complementation experiments demonstrated that it is due to the enclosed *LDOX* gene. In LAB, this defective gene on Ch10 encodes a protein that is ~5% shorter than its counterparts in QLD and other sequenced Solanaceous species, and it also has a unique variant amino acid in a highly conserved domain. A traditional bulk segregant analysis utilises DNA pools from both extreme phenotypes (e.g., a DNA pool from the strong purple response plants and a separate DNA pool from the necrotic response plants), but the difficulty in visually differentiating strong purple (homozygous) from moderate (heterozygous) purple individuals led us to focus solely on plants showing the necrotic response. This single‐pool approach halved the sequencing costs and its success indicates that it may be useful in other situations when one phenotypic extreme is unavailable, such as for an induced lethal trait (Zou et al. 2016). The method also seems to be robust enough to accommodate some contamination since, despite our care in selecting the 40 necrotic phenotype plants to pool, we subsequently discovered it had included 5 contaminating individuals.

There are full‐length and fragmented versions of *LDOX*‐like genes (also referred to as anthocyanidin synthase in some species) on chromosomes other than on Ch10 in *N. benthamiana*, and the 2–oxoglutarate‐dependent dioxygenase superfamily is the second largest enzyme family in plants (Kawai et al. 2014). LDOX catalyses the conversion of leucoanthocyanidins to anthocyanidins, representing the penultimate step in anthocyanin biosynthesis (Wilmouth et al. 2002). Furthermore, there are many other genes involved in the anthocyanin pathway. With these complications, a candidate gene approach to identify the causal gene of the differing anthocyanin response between LAB and QLD would likely have been very time‐consuming, cumbersome and expensive. The BenthMap workflow identified the causal gene region rapidly and could have done so with as little as 10× genome‐wide coverage (an average of 0.25× per individual). Nevertheless, the resolution of the approach also depends on the number of individuals represented in the mapping population, with larger populations resulting in greater precision (James et al. 2013; Wilson‐Sanchez et al. 2019). Increasing the number of selected individuals in the pool (> 40) would likely have further improved the genetic resolution and perhaps facilitate the use of a smaller window size.

To the best of our knowledge, this is the first report of using bulk segregant analysis and mapping by sequencing in *N. benthamiana* to uncover the genotypic cause, from a background of multiple possible candidates, of a natural variant. Our use of parental chromosome‐level genome assemblies bypassed the need for the exome capture technologies typically required for bulk segregant analysis in repeat‐rich genomes (Henry et al. 2014; Krasileva et al. 2017). It shows that the high‐quality parental reference genomes of LAB and QLD enable cost‐effective SHOREmap‐like genetic mapping within this complex allopolyploid species. We expect this to be an effective approach for many of the traits encoded by an estimated 50% of the original ancient parental homoeologous gene pairs that are now retained as single functional copies (Ranawaka et al. 2023). One such example could be the tomato yellow leaf curl virus (TYLCV) resistance gene that is present in QLD but not in LAB and has been recalcitrant to a conventional candidate gene approach (Hayashi et al. 2024). The BenthMap workflow has the potential to accelerate trait‐to‐gene discovery in *N. benthamiana* for applications in recombinant protein production and synthetic biology. A further example could be mapping the genes underlying trichome density. Plant trichomes are excellent biofactories for metabolic engineering (Chalvin et al. 2020), and QLD exhibits higher trichome density than LAB. Applying BenthMap to identify alleles that increase trichome number in LAB could enable chassis improvements for synthetic biology applications.

## 4 Experimental Procedures

### 4.1 RNAseq Data Analysis

Infiltration assays were carried out to test anthocyanin pigment development using two overexpression vectors: (1) functional *AcMYB110* and (2) an artificially mutagenised dysfunctional version of the gene (control). To generate the dysfunctional *AcMYB110* construct, the *AcMYB110* sequence of the *AcMYB110* overexpression construct (pCAMBIA‐35S:*:AcMYB110*) was subjected to digestion with *Sal*I restriction enzyme (NEB), followed by blunting of the 5′ overhang using T4 DNA polymerase (Fermentas) and circularisation of the plasmid using T4 DNA polymerase (Thermo Scientific). The final construct was confirmed to have a 4 bp insertion at 273 bp of the *AcMYB110* sequence, introducing a premature stop codon to the coding sequence. All plants were infiltrated at approximately 4.5 weeks old. The infiltrated leaf patches were collected at 2, 5 and 7 dpi using a 13 mm cork borer. Three biological replicates, each consisting of three individual leaf patches, were collected for the RNAseq experiment. Tissue samples were immediately frozen in liquid nitrogen and stored at −80°C until RNA extraction.

Total RNA was extracted using the RNeasy Plant Mini Kit (Qiagen) with the addition of on‐column DNase digestion using the RNAse‐Free DNase Set (Qiagen) following the manufacturer's instructions. Quality and integrity of the resulting RNA was assessed using the Fragment Analyser system (Advanced Analytical Technologies Inc.) prior to library preparation at Central Analytical Research Facility (CARF; QUT). Library preparation was carried out using the Illumina Stranded mRNA Prep kit and sequenced on NovaSeq 6000 system (Illumina) with 100 bp paired‐end (PE) reads using the SP Flow Cell. Reads from 36 libraries (Figure S1) were mapped to the reference transcriptomes of LAB and QLD.

Galaxy Australia was used for bioinformatics analyses (https://usegalaxy.org.au/; (Community 2024)). Raw reads obtained from each lane of the flow cell were concatenated using the Concatenate multiple datasets v0.2 tool. Cutadapt v4.4 (Martin 2011) was used to remove adapter sequences (Illumina 3′ adapter sequence CTGTCTCTTATACACATCT), and 1 base from each read was removed before adapter trimming. The minimum read length of 25 bases, and a quality cut off of 20 was used to discard low‐quality reads. FastQC (v0.72; https://github.com/s‐andrews/FastQC) and MultiQC v1.9 were used to check the quality of reads before and after trimming. The Strandness of the library was determined using the tool HISAT2 v2.1.0 (Kim et al. 2019) and Infer Experiment v2.6.4.1 using default parameters. Kallisto quant v0.48.0 (Bray et al. 2016) was used to align reads to the reference transcriptome of LAB v3.60 and QLD v1.83 (https://www.nbenth.com, Ranawaka et al. 2023). Strandness of the library was set as ‘Strand specific reads, first read reverse’ and the number of bootstrap samples was set to 100. DESeq2 v2.11.40.6 (Love et al. 2014) was used to run 12 differential expression analysis tests, summarised in Table S9. For each pairwise comparison, the count file output from Kallisto for three biological replicates per treatment was used. The result file output from DESeq2 was filtered to only retain differentially expressed genes with a *p*‐value < 0.001 and absolute log2 (fold change) > 1.

### 4.2 Plant Materials and Phenotyping

A biparental F1S1 mapping population was generated by manual cross‐pollination of *N. benthamiana* LAB and QLD accessions, as previously described by Bally et al. (2015) and Ranawaka et al. (2023). The resulting F1 seeds were grown to self‐fertilise to obtain F1S1 (also known as F2) seeds. Seeds of *N. benthamiana* LAB and QLD accessions, F1 and F1S1, were grown in a soil mix (UQ23; 30% coco peat, 70% composted pine bark) supplemented with Scotts Osmocote controlled release fertiliser (5 mL/L soil), in a controlled environment at 25°C, day length of 16 h and relative humidity at 60%–70%.

4 week old plants were used for transient agroinfiltration with pHEX‐35S::*AcMYB110* as previously described (Hellens et al. 2005). Two leaves per plant were infiltrated with *Agrobacterium tumefaciens* (GV3101 strain) carrying the pHEX‐35S::*AcMYB110* vector at an OD_600_ of 0.1. Infiltrated leaf patches were visually observed for phenotyping at 5 dpi. Fresh young leaves from each individual were collected and labelled according to the phenotype of their infiltration patch and stored individually at −80°C until subsequent DNA extraction.

### 4.3 DNA Extraction and Quantification

For mapping the allele responsible for the necrotic phenotype in LAB, 40 F1S1 individuals with the necrotic phenotype were selected for mapping. DNA was extracted from each plant using the CTAB method or DNeasy plant mini kit (Qiagen) and quantified using NanoDrop spectrophotometer and Qubit fluorometer (Thermo Fisher Scientific). Before pooling individual DNA samples, the quality of DNA was confirmed by NanoDrop (A260/280 > 1.8 and A260/230 ≈2) and by agarose gel electrophoresis. An equal amount of DNA from each of the 40 necrotic phenotype individuals was mixed in a single tube to represent the sequencing pool (Table S1). The pooled DNA was cleaned using MaXtract (Qiagen) and Ampure bead (Beckman Coulter) following the manufacturer's instructions and was preserved in DNAstable (Biomatrica) for transport. Sequencing was carried out at Lincoln Genomics, Lincoln University New Zealand, using a MGI‐DNBG400 sequencer, producing 150 bp paired‐end reads.

### 4.4 Gene Mapping Using BenthMap

Genome and annotation of *N. benthamiana* LAB (v3.60) and QLD (v1.83) were obtained through the *Nicotiana benthamiana* genome website (https://benthgenome.qut.edu.au/; Ranawaka et al. 2023).

The sequence reads were trimmed using fastp (v0.23.4, Chen et al. 2018). FastQC (v11.7; https://github.com/s‐andrews/FastQC) was used for determining quality and GC content. Forward reads from four lanes were combined into one file, and the same was carried out for all reverse reads. Cleaned reads of the F1S1 pool were aligned to either the LAB or QLD genome using Burrows‐Wheeler Aligner (BWA; Li and Durbin 2009), followed by conversion to BAM, sorting and indexing using SAMtools software (settings; −Bh for converting to BAM) to generate 19 BAM files, one for each chromosome. Additionally, read mapping statistics were analysed using samtools idxstats. Variant calling was carried out using samtools mpileup (Danecek et al. 2021) and VarScan v2.4.6 (Koboldt et al. 2012). The resulting vcf files were filtered to select four columns (1, 2, 5 and 19) containing ‘chromosome’, ‘position’, ‘alternate allele’ and ‘percentage of reads with alternate allele’. Furthermore, numbers of homozygous, heterozygous and borderline SNPs were calculated using awk commands. For selecting variants within coding regions only, VCFannotate and VCFfilter (Garrison et al. 2022) within the Galaxy Australia platform (https://usegalaxy.org.au/; Community 2024) were used to filter variants found in coding regions only. A custom perl script (Gardiner et al. 2014) was used to calculate the homozygosity score. The script was generously provided by Dr Laura‐Jayne Gardiner. In this method, SNPs are categorised as homozygous, heterozygous or borderline based on the percentage of F1S1 reads having an alternate base. The SNP calls derived from VarScan were categorised as a homozygote (alternative base in ≤ 100% and ≥ 80% of reads), borderline (alternative base in < 80% and > 70% of reads) and heterozygote (alternative base in ≤ 70% and ≥ 30% of reads). This was followed by a custom R script to plot the resulting homozygosity scores for each chromosome. The bioinformatic pipeline was run for F1S1 reads aligned to each of the parental genomes (LAB and QLD) separately.

To determine heterozygosity of LAB and QLD genomes (Table S10), tissue‐specific (flower, leaf, root, stem, and seedling) paired‐end RNA sequencing data were pre‐processed using fastp (v0.23.4, Chen et al. 2018). All RNAseq reads were pooled into a single set of paired‐end FASTQ files. The STAR aligner (v2.7.10a, Dobin et al. 2012) was used to map the pooled reads to the LAB and QLD genomes using the two‐pass alignment mode to improve splice junction detection.

SNPs were identified from the resulting BAM files using the custom tool bamCoverage (https://github.com/anjiyuan/bamCoverage). This resulted in chromosome‐level summaries of read coverage, insertions, deletions, and mismatches. To focus on coding regions, the codingRegion_SNP module was used in conjunction with gene annotations in GFF3 format. This module integrates mismatch data with coding region annotations to quantify both homologous and heterozygous SNPs within annotated coding sequences.

### 4.5 Downsampling of F1S1 Reads

Downsampling of reads was carried out using the fadso (FAstq DownSampling Optimizer) tool (Kuwahara and Gao 2023). Fadso was used to reduce the number of reads to 50×, 20×, 10× and 5× coverage of the *N. benthamiana* genome. The number of paired F1S1 reads required to achieve a desired depth of coverage was calculated using the formula: coverage = (read count × read length)/total genome size. The number of F1S1 reads used to achieve different depths of coverage is listed in Table S5. The downsampled fastq files were re‐analysed as described in Figure 2, to identify the optimal coverage required to identify the region of interest.

### 4.6 Fine Mapping of Causal Gene Using Molecular Markers

Genomic DNA from 40 F1S1 individuals exhibiting the necrotic phenotype (same individuals used for BSA), and 23 individuals having the purple phenotype were used for fine mapping. Five molecular markers were investigated using primer pairs spanning an InDel or SNPs between LAB and QLD as described in Table S8. The molecular markers were developed by exploiting SNPs identified within the target region as determined by homozygosity scoring. RFLP markers were identified using alignment files (BAM files) by performing manual inspection of the target region in IGV software (Robinson et al. 2011).

### 4.7 Cloning and Overexpression of LDOX Genes

The pS191 (empty vector) obtained from Waterhouse LAB, QUT (Figure S6) was used to construct the LDOX overexpression vectors. The full length coding sequence of each of the six LDOX homologues was amplified using Phusion High‐Fidelity DNA PCR Master Mix (New England Biolabs; NEB) using genomic DNA from LAB or QLD as template, and gene specific primers containing *Sal*I and *Not*I recognition sequences in the forward and reverse primers, respectively, were used for amplification. Each LDOX gene was individually cloned into pS191 at 3′ of the 35S promoter by restriction digest cloning using *Sal*I and *Not*I and sequence verified by Sanger sequencing before being transformed into *A. tumefaciens* (GV3101).

Overexpression of LDOX genes was carried out by agroinfiltrating either the LDOX overexpression vector alone, 1:1 mixture of the LDOX overexpression vector and pS191 (empty vector) or a 1:1 mixture of the LDOX overexpression vector and AcMYB110.

Sequences of primers used for cloning and sequencing are provided in Table S8.

To construct the cladograms, nucleotide sequences of coding regions of LDOX genes were aligned using MUSCLE v5.1 (Edgar 2021), then IQ‐TREE v3.0.1 (Minh et al. 2020; Wong et al. 2025) was used to estimate the best fit substitution model using the MFP algorithm (Kalyaanamoorthy et al. 2017), and the branch support values were obtained using ultrafast bootstrap approximation (UFboot) (Hoang et al. 2017). The cladogram was annotated and visualised using iTOL (Letunic and Bork 2021).

## Author Contributions

P.M.W., J.B., S.H., C.W., J.A. and Z.A. conceived and designed the research. Z.A., S.H., C.W., J.B., J.A. and P.W. conducted the experiments and data analysis. Sequencing was carried out by Z.A., S.H., J.B., P.M.W., C.W. and J.A. wrote the manuscript.

## Funding

This work is supported by a QUT Postgraduate Research Award to ZA. This work was funded by The Australian Research Council Centre of Excellence for Plant Success in Nature and Agriculture Award CE200100015 to PW. Lincoln University funded the whole genome sequencing. This work is supported by Galaxy Australia, a service provided by Australian BioCommons and its partners. The service receives NCRIS funding through Bioplatforms Australia, as well as Queensland Government RICF funding. Computational (and/or data visualisation) resources and services used in this work were provided by the eResearch Office, Queensland University of Technology. Figures were created in BioRender (https://BioRender.com).

## Conflicts of Interest

The authors declare no conflicts of interest.

## Supporting information


**Figure S1:** Phenotype of LAB and QLD at three different time‐points selected for RNAseq (T1 (2dpi), T2 (5dpi) and T3 (7dpi)), after treatment (AcMYB110 or control infiltration) (top panel) and list of samples obtained to generate 36 RNAseq libraries (bottom panel).
**Figure S2:** Heatmaps showing expression patterns of genes involved in anthocyanin biosynthesis.
**Figure S3:** Analysis of significantly differentially expressed genes in LAB and QLD over three time‐points (T1, T2 and T3), representing total genes and upregulated/downregulated genes in AcMYB110 leaf compared to Control leaf.
**Figure S4:** Venn diagrams comparing significantly differentially expressed genes (p‐value < 0.001) at three time‐points (Day 2, 5 and 7) in LAB infiltrated with AcMYB110, and re‐analysed data obtained from Bond et al. (2016) where LAB leaf was infiltrated with AcMYB10 and sampled at 3 days post infiltration.
**Figure S5:** Sequence alignments of LDOX/ANS proteins. Sequence similarity and identical residues seen in Iron(II)‐Dependent Oxygenases are shown as yellow and orange highlights, respectively.
**Figure S6:** pS191 vector map.
**Figure S7:** Effect of transient co‐expression of LAB and QLD LDOX homologues and AcMYB110 on anthocyanin accumulation in LAB and QLD leaf. Pictured at 5 days after infiltration with; (M) 35S::*AcMYB110*, (C) PS191 (empty vector) as a control, (1) 1:1 mixture of 35S::*AcMYB110* + 35S::*QLD‐Ch10‐LDOX*, (2) 1:1 mixture of 35S::*AcMYB110* + 35S::*LAB‐Ch10‐LDOX*, (3) 1:1 mixture of 35S::*AcMYB110* + 35S::*QLD‐Ch17‐LDOX*, (4) 1:1 mixture of 35S::*AcMYB110* + 35S::*LAB‐Ch17‐LDOX*, (5) 1:1 mixture of 35S::*AcMYB110* + 35S::*QLD‐Ch04‐LDOX*, and (6) 1:1 mixture of 35S::*AcMYB110* + 35S::*LAB‐Ch04‐LDOX*. Scale bar represents 1 cm.

**Table S1:** Concentration and absorbance of individual DNA samples pooled for whole genome resequencing.
**Table S2:** Summary of FastQC results for raw data and cleaned data (after trimming using fastp).
**Table S3:** Summary of read mapping statistics of F1S1 reads aligned to the LAB or QLD genome.
**Table S4:** Numbers of SNPs obtained for F1S1 reads aligned to LAB or QLD genomes, including all SNPs (genomic) and those within coding regions only (CDS).
**Table S5:** Number of paired reads corresponding to coverage of the *N. benthamiana* genome.
**Table S6:** List of significantly differentially expressed genes (adjusted *p*‐value < 0.001) in response to AcMYB110 overexpression, within the 3.5 Mb candidate region of the QLD genome including NbQ17g26695.
**Table S7:** List of significantly differentially expressed genes (*p*‐value < 0.001), in response to AcMYB110 overexpression, within the region on the LAB genome corresponding to the 3.5 Mb candidate region of the QLD genome, including NbL17g25680 and NbL04g00685.
**Table S8:** List of primers used in this study.
**Table S9:** Summary of pair‐wise differential expression analysis.
**Table S10:** Numbers of SNPs obtained for LAB and QLD RNAseq reads aligned to LAB or QLD genomes.
**Table S11:** Genbank accession numbers, sizes and source of LDOX/ANS sequences.
**Table S12:** CDS sequences of *N. benthamiana* LDOX genes.

## Data Availability

The data that support the findings of this study are openly available in NCBI Sequence Read Archive (SRA) at https://dataview.ncbi.nlm.nih.gov/object/PRJNA1309167?reviewer=u3ff94pqp7g4bp5egno6fgg86u, reference number PRJNA1309167.
