# Supplementary material for: Harnessing Bulk‐Segregant Mapping to Identify Trait‐Associated Genes in the Allopolyploid Model Plant Nicotiana benthamiana
Source: Plant Biotechnol J. 2026 Jan 29;24(11):5575–87. doi: 10.1111/pbi.70560 (PMC13398463; doi:10.1111/pbi.70560)
Supplement: Supplementary file 1 [file PBI-24-5575-s002.docx]

Harnessing bulk-segregant mapping to identify trait-associated genes in the allopolyploid model plant *Nicotiana benthamiana*

Zuba Ahmed^1, 2^, Jiyuan An^1, 2^, Satomi Hayashi^1, 2^, Julia Bally^1, 2^, Chris Winefield^3, 2^, Peter Waterhouse^1, 2^

*1. Centre for Agriculture and the Bioeconomy, School of Biology and Environmental Sciences, Queensland University of Technology, Brisbane City, QLD, Australia*

*2. Centre of Excellence for Plant Success in Nature and Agriculture, Queensland University of Technology, Brisbane City, QLD, Australia*

*3. Department of Wine Food and Molecular Biosciences, Faculty of Agriculture and Life Sciences, Lincoln University, Te Whare Wānaka o Aoraki, Lincoln, Christchurch, New Zealand*

**Supplementary Figures**

**Figure S1.** Phenotype of LAB and QLD at three different time-points selected for RNAseq (T1 (2dpi), T2 (5dpi) and T3 (7dpi)), after treatment (AcMYB110 or control infiltration (top panel) and list of samples obtained to generate 36 RNA-seq libraries (bottom panel).

**Figure S2** Heatmaps showing expression patterns of genes involved in anthocyanin biosynthesis. White to blue to red colour indicates increasing levels of expression in scaled log10 transcripts per million (TPM). Analysis of reads aligned to the LAB v3.60 transcriptome.

**Figure S3**. Analysis of significantly differentially expressed genes in LAB and QLD over three time-points (T1, T2 and T3), representing total genes and upregulated/downregulated genes in *AcMYB110* leaf compared to Control leaf. Alignment of both LAB and QLD reads to LAB transcriptome (solid bars) is compared to the QLD transcriptome (dashed bars). Venn diagrams displaying comparisons between the number of significantly differentially expressed genes (p-value < 0.001) at three time-points in LAB and QLD. Alignment of both LAB and QLD reads to LAB transcriptome (top) is compared to alignments to the QLD transcriptome (bottom).

**Figure S4.** Venn diagrams comparing significantly differentially expressed genes (p-value < 0.001) at three time-points (Day 2, 5 and 7) in LAB infiltrated with *AcMYB110*, and re-analysed data obtained from Bond et al., (2016) where LAB leaf was infiltrated with *AcMYB10* and sampled at 3 days post infiltration. All DEGs were identified from alignments to the LAB v3.60 transcriptome.

**Figure S5**. Sequence alignments of LDOX/ANS proteins. Sequence similarity and identical residues seen in Iron(II)-Dependent Oxygenases are shown as yellow and orange highlights, respectively. Secondary structure assignments including α helices and β strands, are shown as blue helices and green arrows, respectively. Iron binding residues are indicated by empty triangles. Residues involved in binding 2OG, DHQ-1, DHQ-2 and MES/ascorbate are shown in purple, pink, red and grey dots. All annotations are according to the *Arabidopsis thaliana* ANS crystal structure (Wilmouth et al., 2002). Purple box highlight indicates the G131R mutation in LAB-Ch10-LDOX. The DIOX_N domain (Interpro: IPR026992) is from position 54 to 167.

**Figure S6**. pS191 vector map

**Figure S7**. Effect of transient co-expression of LAB and QLD LDOX homologues and *AcMYB110* on anthocyanin accumulation in LAB and QLD leaf. Pictured at 5 days after infiltration with; (M) 35S::AcMYB110, (C) PS191 (empty vector) as a control, (1) 1:1 mixture of 35S::AcMYB110 + 35S::QLD-Ch10-LDOX, (2) 1:1 mixture of 35S::AcMYB110 + 35S::LAB-Ch10-LDOX, (3) 1:1 mixture of 35S::AcMYB110 + 35S::QLD-Ch17-LDOX, (4) 1:1 mixture of 35S::AcMYB110 + 35S::LAB-Ch17-LDOX, (5) 1:1 mixture of 35S::AcMYB110 + 35S::QLD-Ch04-LDOX, and (6) 1:1 mixture of 35S::AcMYB110 + 35S::LAB-Ch04-LDOX. Scale bar represents 1 cm.

BOND, D. M., ALBERT, N. W., LEE, R. H., GILLARD, G. B., BROWN, C. M., HELLENS, R. P. & MACKNIGHT, R. C. 2016. Infiltration-RNAseq: transcriptome profiling of Agrobacterium-mediated infiltration of transcription factors to discover gene function and expression networks in plants. *Plant Methods,* 12**,** 41.

WILMOUTH, R. C., TURNBULL, J. J., WELFORD, R. W. D., CLIFTON, I. J., PRESCOTT, A. G. & SCHOFIELD, C. J. 2002. Structure and Mechanism of Anthocyanidin Synthase from Arabidopsis thaliana. *Structure,* 10**,** 93-103.
